# Synergistic/Antagonistic Potential of Natural Preparations Based on Essential Oils Against *Streptococcus mutans* from the Oral Cavity

**DOI:** 10.3390/molecules24224043

**Published:** 2019-11-07

**Authors:** Vlad Tiberiu Alexa, Atena Galuscan, Iuliana Popescu, Emil Tirziu, Diana Obistioiu, Alin Daniel Floare, Antonis Perdiou, Daniela Jumanca

**Affiliations:** 1 Faculty of Dental Medicine, “Victor Babeş” University of Medicine and Pharmacy, Eftimie Murgu Sq. No. 2, 300041 Timişoara, Romania; vlad_alexa94@yahoo.com (V.T.A.); alinfloare@yahoo.com (A.D.F.); aperdiou@gmail.com (A.P.); jumanca.daniela@umft.ro (D.J.); 2 Faculty of Agriculture, Banat’s University of Agricultural Sciences and Veterinary Medicine “King Michael I of Romania” from Timişoara, Calea Aradului No. 119, 300641 Timişoara, Romania; iuliapopescu2002@yahoo.com; 3 Faculty of Veterinary Medicine, Banat’s University of Agricultural Sciences and Veterinary Medicine “King Michael I of Romania” from Timişoara, Calea Aradului No. 119, 300641 Timişoara, Romania; emiltirziu@yahoo.com (E.T.); diana.obistioiu@yahoo.com (D.O.)

**Keywords:** gas chromatography-mass spectrometry (GC-MS), orange essential oil, bergamot essential oil, cloves essential oil

## Abstract

The present paper addresses a thematic of interest in preventive dental medicine, namely the possibility of using essential oils (EOs) for the inhibition of the development of *Streptococcus mutans* (*S. mutans*) in the oral cavity, as a viable alternative to chemical products with protective role in oral health. For this purpose, four EOs (cinnamon, clove, bergamote, and orange) were chemically characterized by gas chromatography coupled with mass spectrometry (GC-MS) and in vitro tested against *S. mutans* (ATCC 25175). The results obtained revealed the antibacterial effect on *S. mutans* exercised by the essential oils of clove (CLEO), bergamote (BEO), and orange (OEO), which were included in the production of natural emulsion-type preparations with application in dental medicine. In order to highlight the synersistic/antagonistic effects generated by the chemical constituent of essential oils, binary and tertiary emulsions were prepared and used in saliva-enhanced medium against *S.* *mutans*. The saliva tests proved the synergistic effect exercised by the active components of EOs tested from tertiary emulsions, which cause an inhibition of the development of *S. mutans* in oral cavities.

## 1. Introduction

In a living organism, dental integrity is constantly threatened by microorganisms; hence, dental caries represent one of the most widespread medical conditions. According to the literature, dental caries rank third in terms of medical costs, outranked only by heart disease and cancer [1]. Although the oral cavity of most mammalian species contains billions of microbial cells, scientific research has shown that *Streptococcus mutans* (*S. mutans*) is the bacteria with the highest responsibility for the appearance of dental caries [2].

Several factors such as adhesion to dental enamel surfaces, production of acid metabolites, ability to synthesize glycogen stores, and ability to synthesize extracellular polysaccharides, mean *S. mutans* plays a central role in the etiology of dental caries [3]. Together with *Lactobacillium* species, *S. mutans* are strongly acid-producing bacteria that cause the risk of dental caries [4]. Usually, the presence of *S. mutans* on the teeth surface is followed within 6–24 months by the appearance of dental caries [5].

In dentistry, the use of plants as an adjunct in the prevention and treatment of various gingival or paradontal diseases reveals their antibacterial role. Previous studies highlighted the potential beneficial effect of extracts or essential oils (EOs), including citrus oils, in treating bacterial or fungal diseases located in the oral cavity [6]. Citrus fruits were cultivated in an ever-widening area since ancient times and exhibit well-documented nutritional and health benefits [7,8]. 

Orange oil (OEO) is an essential oil produced by the cells of the orange or sweet oranges (*Citrus Sinensis*) fruit, one of the 30 species of Citrus genus belonging to the Rutaceae family. The chemical composition of OEO is influenced by several factors such as genetic differences between varieties and species, environmental factors, maturity level, and harvesting practices [9]. The main chemical compounds of the OEO are limonen, alpha-pinen, sabinen, β-pinen, mycrene, linalool, citronelal. OEO has many traditional ethnomedicinal benefits such as treating spasms, sedative effects, reducing inflammation and preventing infection, reducing depressive states, improving immunity, improving cognitive functions in Alzheimer’s disease, and also in vitro antimicrobial activity [10].

Bergamot fruit (*Citrus aurantium* L.), belonging to the Citrus genus family *Rutaceae* is widespread in the Mediterranean ecoregion, especially in the southern part of Italy and Greece. Genetic research in the ancestral origins of this citrus found that it is a hybrid between bitter orange and lemon [11]. Historically, bergamot fruit was used in Italian popular medicine to treat malaria, to reduce fever, and as an antiseptic. The current production of bergamot oil (BEO) from the coastal region of Italy, in the Calabria region, accounts for 80% of the world’s total production and is considered to be of the highest quality on the international market. Its volatile oil finds application in a wide range of perfumes, cosmetics, and especially in aromatherapy [12].

In addition, cloves as a spice, especially in the gastronomy of countries in Asia, Africa, and the Middle and Far East, are important herbs due to the wide range of pharmacological effects consolidated in traditional use for centuries [13]. Previous studies present the most important biological qualities of cloves essential oil (CLEO) and the main compound (eugenol): Antioxidant, antimicrobial, antiviral, and antinociceptive effect, especially in dental, articular, and antispasmodic pain [13]. The antibacterial activity of CLEO has been demonstrated on several bacteria and fungal strains [14,15]. CLEO is generally devoid of toxicity when consumed at concentrations less than 1500 mg/kg. The World Health Organization (WHO) has established the acceptable daily amount of CLEO per day as 2.5 mg/kg in humans [14]. The use of CLEO can, therefore, be a viable alternative as an antimicrobial agent, as it is a natural, cheap, and effective method of controlling bacteria involved in oral infections.

The term cinnamon usually refers to the dry bark of *C. Zeylanicum* (also known as cinnamon in the Sri Lanka area) and *C. Aromaticum* (also known as Chinese cinnamon) used for the preparation of different types of chocolate, drinks, and liqueurs. In Traditional Chinese medicine (dating about 4000 years), cinnamon has been used as a neuroprotective agent and for the treatment of diabetes. Cinnamon has also been used as a health promotion agent for the treatment of diseases such as inflammation, gastrointestinal disorders, and urinary infections. Another potential use of medical cinnamon would be its antibacterial role. One of the most popular properties of cinnamon extracts and essential oil of cinnamon and their components is the antibacterial activity against Gram-positive and Gram-negative bacteria responsible for human infectious diseases and the degradation of food or cosmetics [16]. In the field of dental medicine, studies were conducted certifying the antibacterial properties of cinnamon on S. *mutans* [17].

The aim of this paper is to study the antibacterial potential of essential oils of cloves (CLEO), cinnamon (CIEO), oranges (OEO), and bergamot (BEO) against *S. mutans* from the oral cavity in the proliferation of dental caries. The antibacterial effects of natural products are exerted by the chemical constituents; for these reasons, it is important to know the composition of active principles of essential oils and their role in inhibition of bacterial growth. Therefore, the composition of the essential oils was studied by gas chromatography coupled with mass spectrometry (GC-MS) in the first step. The antibacterial potential required in vitro testing of the individual oils and of the main chemical compounds by determining the optical density (OD) in the presence of *S. mutans* inoculation. EOs that have proven to be effective, wereincluded in natural preparations in varying ratios, and the antibacterial effects induced by synergistic and/or antagonistic effect of the chemical compounds were tested under the same in vitro conditions on *S. mutans*. Further, for use in dentistry practice, preparations with optimal activity weretested by applying treatments at different concentrations to biological samples of saliva inoculated with *S. mutans.*

The novelty of this study consisted of synersistic/antagonistic effects exerted by the use of binary or tertiary emulsions based on essential oils (EOs) with applications in dentistry. 

## 2. Results and Discussion

### 2.1. GC-MS Composition of EO_S_

The chemical composition of the analyzed EOs is presented in Table 1. The results show that CLEO contains eugenol as the major component (80.11%), followed by eugenol acetate (13.54%), while CIEO has cinamil-alcohol as a major compound (88.45%). In BEO, 14 volatile compounds were identified, of which D-limonene represents 49.38% and α-Pinene 32.63% of the total components. The analyzed OEO contains 97.93% D-limonene.

Monoterpene hydrocarbonates (MH) are the major compounds in BEO (96.524%) and OEO (99.45%), while monoterpene oxygenate (MO) are found in CLEO (94.44%) and in CIEO (97.948%). The results obtained for the chemical composition of the EOs are in agreement with those reported in the literature [18,19,20,21,22,23,24,25]. Mohagheghniapour et al. (2018) identified the following composition of orange essential oil: Linalool acetate (12.2 ± 0.08%–28.9 ± 0.2%), linalool (22.9 ± 0.07%–54.0± 0.2%), farnesol (0.2 ± 0.04%–10.4 ± 0.07%), E-nerolidol (0.4 ± 0.1%–21.4 ± 0.04%), and geranyl acetate (0.97 ± 0.05%–9.3 ± 0.08%) [18]. Noteworthy, significant differences in the content of D-limonene (1%–14%) and β-pinene (0%–9.6%) were observed depending on the extraction method applied [19].

Previous studies have shown that limonene is the main chemical component of orange essential oil (68%–98%), while in bergamot essential oil, the concentration of limonene varies between 30%–40% [20]. Similar results were reported by Azar et al. and Tao et al., in 2011 and 2009, respectively. Both studies identified limonene as the major component in the essential oil of sweet orange peel (77.49%), followed by myrcene (6.27%), α-farnesene (3.64%), and γ-terpinene (3.34%) [21,22]. Also, α-pinene (0.5% and 2.4%) and linalool (1.2% and 0.9%) were identified in reduced concentrations [23]. In bergamot essential oil, D-limonene (60.44%) and γ-terpinene (20.28%) were the major compounds identified [24], while in the study by Verma et al. in 2016, linalool (33.9%–77.1%) and linalyl acetate (2.2%–45.4%) were identified in different percentages [25].

In the case of cinnamon leaf essential oil, the main component is eugenol, which reaches a concentration of over 80%, while CLEO contains approximately 89% eugenol and 5% to 15% eugenol acetate and β-caryophyllene [16]. 

The chemical composition of EOs is influenced by several factors, such as genetic differences between varieties and species, environmental factors, maturity level, and harvesting practices [9].

### 2.2. In Vitro Antibacterial Activity of EOs and of Main Chemical Compounds against S. mutans

**Figure 1:**
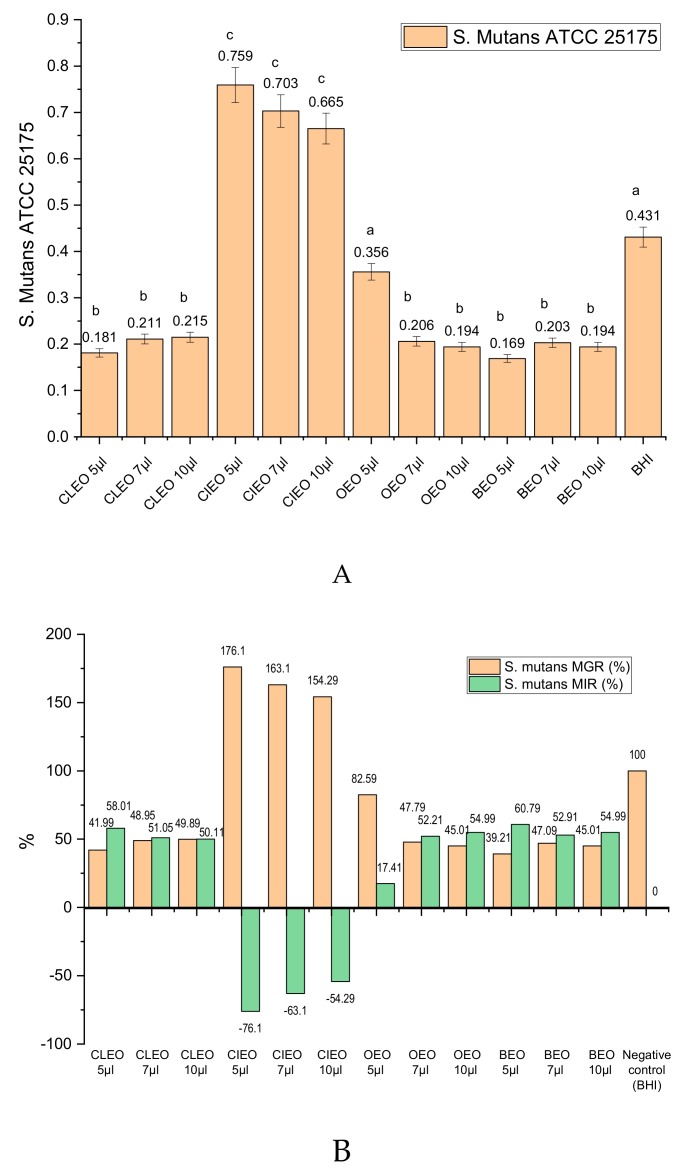
Antibacterial activity of essential oils (EOs) against *S. mutans*. (**A**) 540 OD values (oils with antibacterial activity have optical density (OD) values < negative control); different letters in columns indicate significant differences between values according to the *t*-Test (*p* < 0.05) after analysis of variance (ANOVA one-way) (**B**) Micellium growth rate (MGR) (%) and micellium inhibition rate (MIR) (%) for the essential oils analyzed in different concentrations.

Figure 1A shows the experimental results of the antibacterial activity expressed as optical density (OD at 540 nm) of the analysed oils (CIEO, CLEO, BEO, and OEO) and negative control brain heart infusion (BHI), after incubation for 24 h on *S. mutans*. It can be observed that the OD values in the case of CIEO use in different concentrations are higher than the negative control represented by the BHI suspension without the addition of EO. The other three EOs analyzed show effects as a result of the inhibition of the development of *S. mutans* (lower OD values as negative control). The OD values recorded for CLEO application varies between 0.181 ± 0.007 and 0.215 ± 0.007). In the case of BEO, an inhibition of *S. mutans*’ micellian development for the three applied concentrations (OD value between 0.169 ± 0.006 and 0.203 ± 0.006) was observed; the inhibition being lower when using OEO (OD value between 0.1945 ± 0.008 and 0.356 ± 0.006). As the biomass of the microorganisms is higher, the OD will be higher, i.e., the microorganism inhibition capacity will be smaller. The statistical analysis shows that there are significant differences between the values obtained in the case of the use of EOs and the negative control, for all the variants used. 

The minimum inhibition concentration (MIC) of EOs was determined as being the lowest concentration of the compound that inhibited the visible growth of cells and was established at 5 µL for OEO, CLEO, and BEO. Lower concentrations have been analyzed (2 µL and 4 µL) but no mass loss effect on the *S. mutans* strain was recorded.For CIEO, the MIC value was higher than the maximum concentration tested (10 µL) and, hence, CIEO has not been used in the natural preparation. 

Expressed as micellium growth rate (MGR, %) and micellium inhibition rate (MIR, %) the values are presented in Figure 1B. It is noted that compared to the negative control, for which MGR (%) is considered to be 100%, for lower micelliar growth rates, a positive MIR (%) is achieved in the case of CLEO (50.11%–58.01%), OEO (17.41%–54.99%), and BEO (52.91%–60.79%). However, CIEO promotes the development of *S. mutans* and negative MIR (%) between 54.29%–76.1%. Essential oils of citrus fruits and fresh extracts can be used to prevent the spread of infections, by using it as an antiseptic with antibacterial role [26,27]. Previous studies have shown the potential beneficial effect of extracts or essential oils in treating bacterial or fungal diseases located in the oral cavity [28,29,30]. In the study by Vaishali and Geetha in 2018, the in vitro antibacterial effect of OEO on *S. mutans* was proven at different concentrations being recommended in maintaining oral health [29]. Chaudhari et al. (2012) evaluated the antibacterial activity of CIEO and CLEO against *S. mutans*, proving their efficacy [13], while the antibacterial activity of CLEO has been demonstrated on several strains of bacteria and fungi [30]. These data are confirmed by the results presented in this study, which highlight the active role of OEO, CLEO, and BEO in inhibiting the development of *S. mutans*.

In order to highlight the chemical compounds responsible for the antibacterial effects of EOs analyzed, three standards of main chemical compounds in EOs were tested, under the same conditions, on *S. mutans*, according to the data obtained from GC-MS analysis and presented in Table 1. Thus, d-limonene (majority compound in OEO 97.3% and BEO 47.38%), α-pinene (32.62% in BEO), and eugenol (80.1% in CLEO) were tested. 

**Figure 2:**
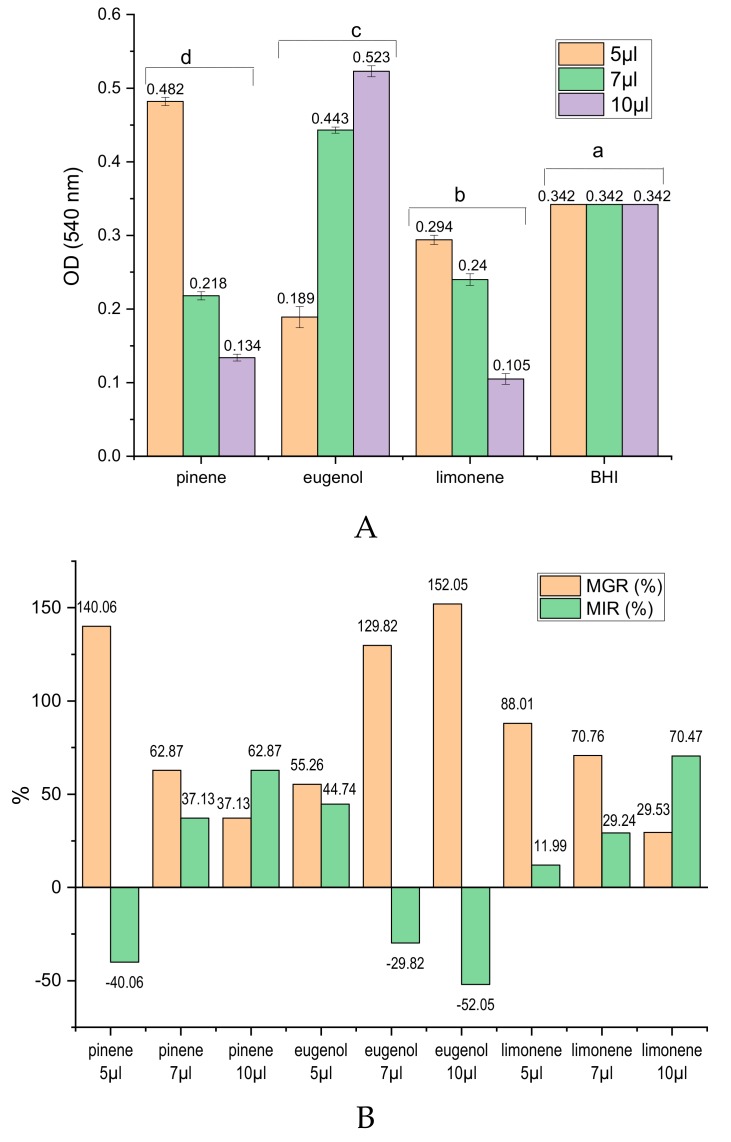
Antibacterial activity of main chemical compounds against *S. mutans*. (**A**) 540 OD values (compounds with antibacterial activity have OD values < negative control); different letters in columns indicate significant differences between values according to the t-Test (*p* < 0.05) after analysis of variance (ANOVA one-way). (**B**) MGR (%) and MIR (%) for the main chemical compounds analyzed in different concentrations.

From the data presented in Figure 2 regarding the antibacterial effects against *S. mutans* of the main chemical components of EOs analyzed, it is observed that limonene and α -pinene have inhibitory capacity of the mycelial development that increases with the standard concentration. OD values lower than the negative control (BHI) were observed for limonene (5, 7, 10 µL) and for α -pinene (7, 10 µL) (Figure 2A). Thus, the MIR for alpha-pinen is 37.13% for 7 µL and 62.87% for 10 µL. An increased inhibition rate shows limonene applied in a concentration of 7 µL (MIR = 29.24%) and 10 µL (MIR = 70.47%), respectively (Figure 2B). On the other hand, eugenol favors the development of *S. mutans* with the increase of the applied concentration, registering high growth rates of *S. mutans* (MGR = 129.82% for 7 µL eugenol and 152.05% for 10 µL of eugenol), relative to negative MIR values (Figure 2B).

The antibacterial capacity of OEO is explained by the activity of limonene, which represents the main chemical compound of this EO. By increasing the concentration of OEO, the content of limonene and the antibacterial activity is more pronounced. In the case of BEO, antibacterial activity is explained by the content of limonene and alpha-pinene from the composition of this oil.

### 2.3. In Vitro Antibacterial Activity of Natural Emulsions Against S. mutans

Based on the results obtained on the antibacterial activity of the EOs tested against *S. mutans*, natural emulsions were prepared for dental use and were tested in vitro on *S. mutans*.The tested concentrations accounted for the effective concentrations established in the in vitro testing of the essential oils were presented in chapter 2.2. 

**Figure 3:**
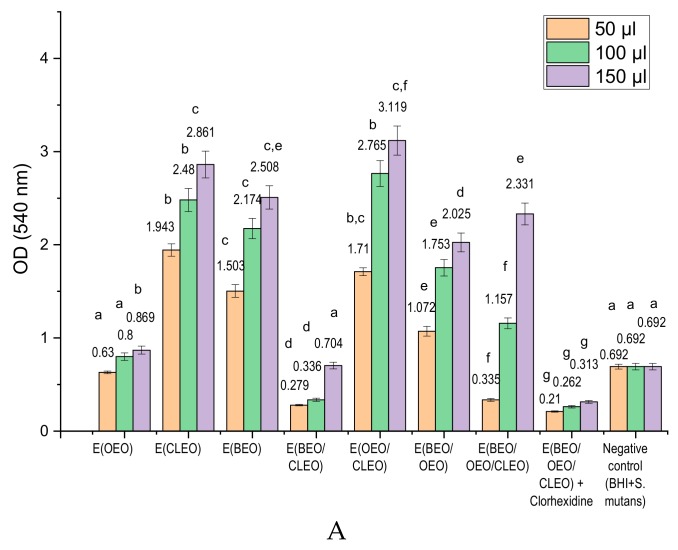

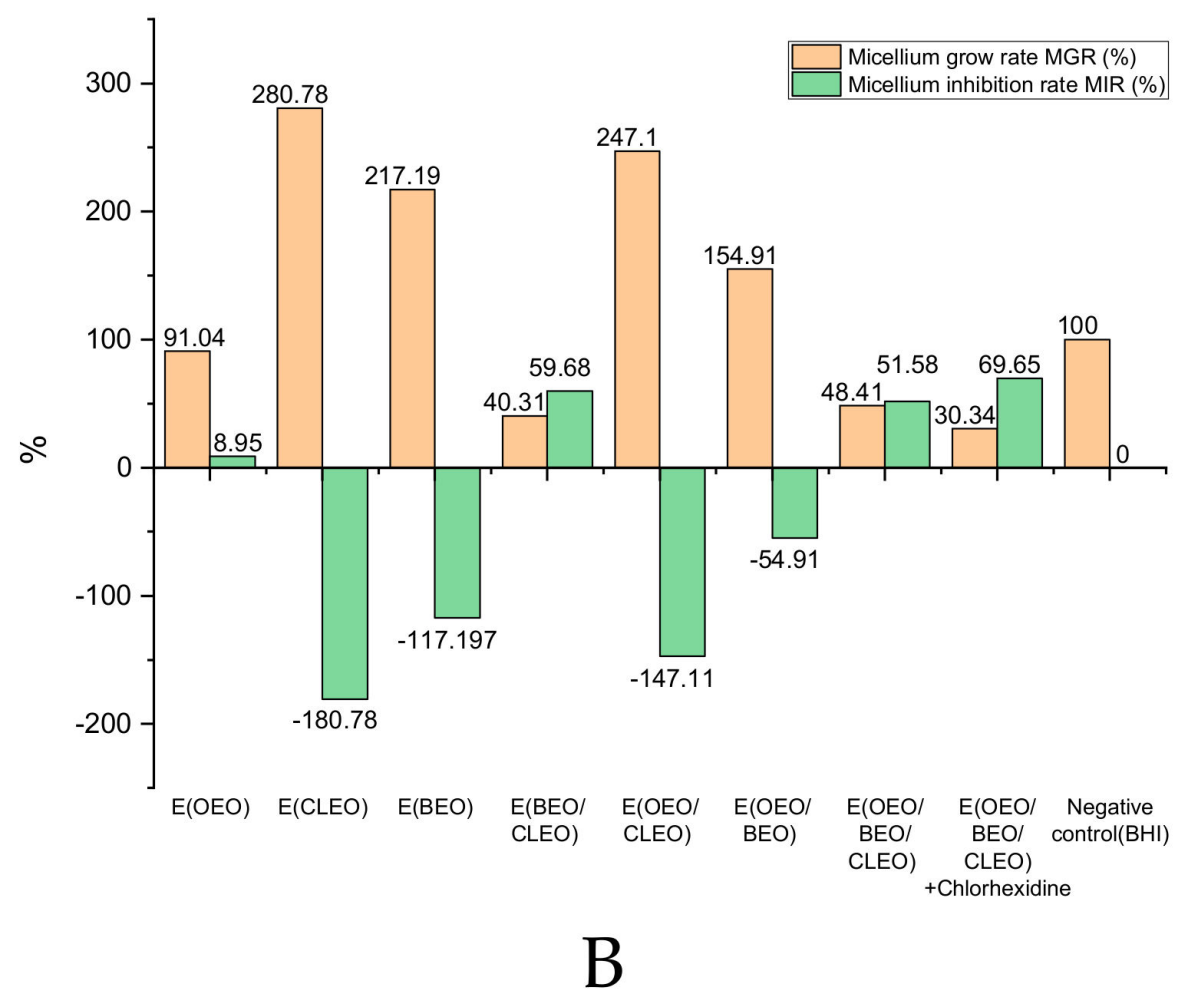
Antibacterial activity of the analyzed emulsions against *S. mutans* (**A**) 540 OD values (emulsions with antibacterial activity have OD values < negative control); different letters in columns indicate significant differences between values according to the t-Test (*p* < 0.05) after analysis of variance (ANOVA one-way). (**B**) MGR (%) and MIR (%) for 50 μL emulsions.

Figure 3A shows the results obtained in testing the analyzed emulsions expressed as OD (540 nm) in relation to the negative control (BHI+*S. mutans*) and the positive control (BHI+*S. mutans*+ Chlorhexidine+tertiary emulsions). It is observed that OD values were higher than the negative control (BHI + *S. mutans*), which indicates a promoting effect of the development of *S. mutans* biomass were recorded for all monoemulsions, even though the corresponding analyzed EOs showed inhibitory effects on the development of *S. mutans* (OD for EOEO, ECLO, and EBEO, between 0.63 ± 0.014 ÷ 0.869 ± 0.018, 1.943 ± 0.066 ÷ 2.861 ± 0.127, and 1.503 ± 0.068 ÷ 2.508 ± 0.113, respectively). The antibacterial activity of the emulsions tested on *S. mutans* (Figure 3B) expressed as MGR (%) and MIR (%) show lower inhibitory effects of the emulsions compared with the corresponding EOs. Positive MIR values, associated with the inhibition process, are recorded when 50 µL binary emulsion E (BEO/CLEO) (MIR = 59.68%) is applied, and for the tertiary mixture E (BEO/CLEO/OEO) with chlorhexidine (MIR = 69.65%) or without chlorhexidine (MIR = 51.58%). 

The effects exerted by the biologically active principles of α-pinene and limonene type are diminished by the presence of the emulsifier (lecithin) and the aqueous environment of the emulsions, the OD values for EBEO being higher than the OD values for BEO. Also, from Figure 2 we can see the potentiation of the micellar development of *S. mutans* by eugenol, a fact confirmed by the results obtained in the case of using ECLEO (Figure 3).

Binary mixture E (BEO/CLEO) is noted to have an important inhibitory effect in all concentrations tested, with the OD values recorded being lower than the negative control. Also, the tertiary mixture E (BEO/OEO/CLEO) applied in a concentration of 50 µL is found to be active in stopping the development of *S. mutans*. The association of the tertiary mixture with chlorhexidine causes maximum inhibitory effects, proven by minimum OD values relative to the negative control. 

The use of natural preparations based on EOs represents viable alternatives to the synthetic chemical preparations used in dentistry. The use of clove oil emulsion in practice has been reported in previous studies. Usage of clove oil nanoformulations in ethanol or with sodium caseinate (NaCas-5%) and pectin (0.1%) as a high-speed coating and homogenization material suggest the possibility of using clove oil as a delivery system for antimicrobial bioactive substances in medicine [31,32,33,34].

In this sense, the present study presents original and novel data by applying binary respective tertiary emulsions based on OEO, CLEO, and BEO with role in teeth protection against dental caries generated by *S. mutans*.

The obtained results revealed that the use of E-type binary emulsions (BEO/CLEO) leads to an inhibition of the microbial mass of *S. mutans*, the inhibitory effect being recorded inversely proportional to the applied concentration. The same enhancing effect of micellar development as the amount of emulsion applied is recorded in the case of binary mixtures of type E (OEO/BEO), as well as of ternary mixtures E (BEO/OEO/CLEO), both in the absence of and in the presence of chlorhexidine. The increase of the inhibitory effect of *S. mutans* in the case of binary or tertiary mixtures of EOs proves the synergistic effect exerted by the active components. The high inhibition effect is recorded for the BEO/CLEO binary mixture. Considering the chemical composition of BEO, which is represented by d-limonene (49.38%) and α-pinene (32.63%), and CLEO, containing mainly eugenol (80.11%), it can be concluded that the combination of these chemical compounds is responsible for the inhibition effect. The association in E (BEO/CLEO) of three major active principles of α-pinene, eugenol, and limonene type causes synergistic effects of potentiation for the antibacterial activity (Figure 3). 

Opposite, the association of eugenol with limonene in the case of binary emulsions E(OEO/CLEO) causes antagonistic effects and favors the development of *S. mutans* mycelium, the same antagonistic effect having the α-pinene and limonene binary combinations in E (OEO/BEO), which suggests the fact that although limonene exerts a strong antibacterial effect, in combination with eugenol or α-pinene separately, the inhibitory effects on *S. mutans* are diminished. 

Last but not least, the study shows that the excipients in the composition of the natural emulsions exert a stimulating effect on the development of *S. mutans*, a fact proven by the increase of the OD values with the increase of the applied emulsion quantity. For the same reasons, binary emulsions based on OEO exert a low antibacterial effect compared to E (OEO). 

The tertiary emulsion E (BEO/CLEO/OEO) applied in concentrations of 50 µL exerts antibacterial effects, but the increase of concentration, respectively of the excipients, leads to the enhancement of *S. mutans* development. 

The use of chlorhexidine in the composition of the tertiary emulsion with maximum inhibition capacity, increases the antibacterial effect on *S. mutans*, due to the synergistic effects obtained. Moreover, chlorhexidine added to the emulsion formed from the ternary mixture of essential oils, diminishes the negative effects of the excipients, causing an inhibition rate higher than the negative control, regardless of the amount of emulsion added.

### 2.4. Antibacterial Activity of EOs and Natural Emulsions Against S. mutans in Saliva Enhanced Medium

**Figure 4:**
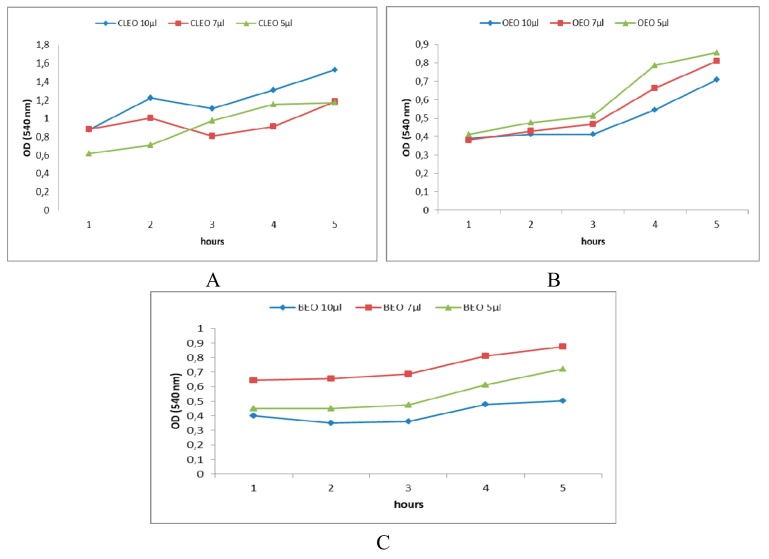
540 nm OD values recorded after 1, 2, 3, 6, and 24 h when using EOs in different concentrations ((**A**)-CLEO, (**B**)-OEO, (**C**)-BEO) as antibacterial agents against *S. mutans*.

**Figure 5:**
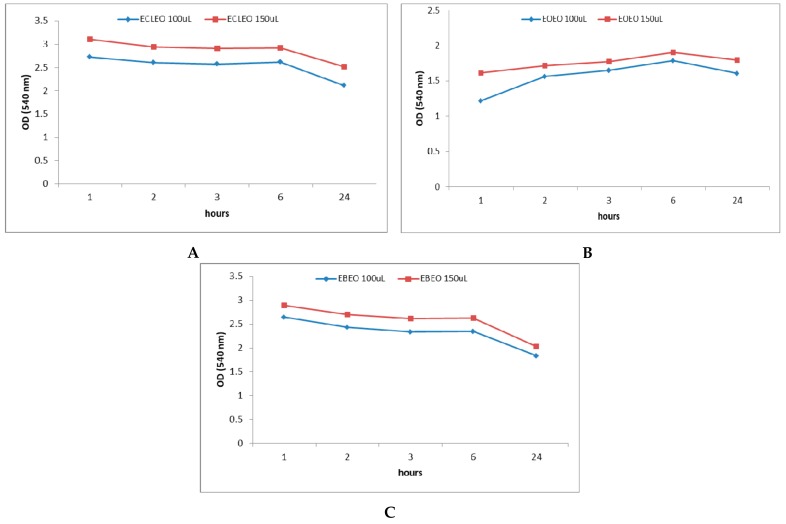
540 nm OD values recorded after 1, 2, 3, 6, and 24 h when using mono emulsions in different concentrations ((**A**)-ECLEO, (**B**)-EOEO, (**C**)-EBEO) as antibacterial agents against *S. mutans*.

**Figure 6:**
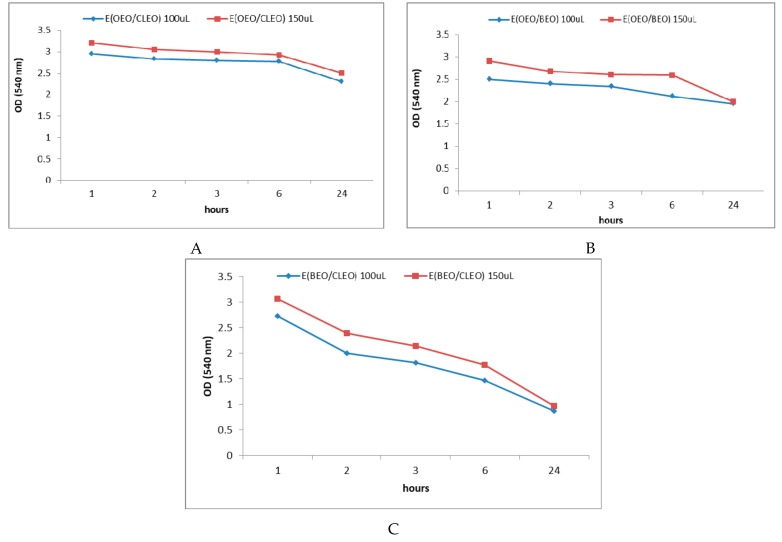
540 nm OD values after 1, 2, 3, 6, and 24 h when using binary emulsions in different concentrations (**A**)-E(OEO/CLEO), (**B**)- E(OEO/BEO), (**C**)-E(BEO/CLEO) as antibacterial agents against *S. mutans*.

Figure 4A–C shows the OD values at 540 nm recorded after 1, 2, 3, 6, and 24 h when using different volumes of EOs as antibacterial agents against *S. mutans* developed in the oral cavity, respectively. Figure 5A–C presents the effect of the obtained emulsions (EOEO, EBEO, ECLEO). Figure 6 shows the OD values obtained over time on the *S. mutans* as a result of the treatment with binary emulsion systems. Figure 6 shows the OD values (540 nm) recorded after 1, 2, 3, 6, and 24 h on *S. mutans* in the case of tertiary emulsions without (Figure 6A) and in the presence of chlorhexidine (Figure 6B). Figure 4A presents OD values for 10 µL of CLEO, which vary between 0.879 after 1 h of inoculation and increases at 1.225 after 2 h. Slight decrease of OD values was recorded after 3 h of inoculation, followed by an increase during 4–24 h. The same profile of the OD curve in time is recorded in the case of OEO and BEO (Figure 4B,C). The minimum value (OD = 0.361), i.e., maximum inhibition efficiency, was attributed to BEO at an applied concentration of 10 µL and 3 h after incubation. 

In the case of CLEO, the increase in the concentration of EO caused a reduction of the antibacterial effect. Based on the results presented in Figure 2 regarding eugenol’s potentation effects on *S. mutans*, we can suggest that this chemical compound of the EOs are responsible for inhibiting the antibacterial effect and by increasing the concentration, the activity of this compound is stimulated.

The effect of using emulsions reported in time emphasizes a relatively constant evolution of OD values during the 24 h of observation (Figure 5). A slight decrease in OD values at 24 h was observed when 100 µL E(BEO) (OD = 2.643 after 1 h and OD = 1.833 after 24 h) and 100 µL E(CLEO) (OD =2.726 after 1 h and OD = 2.144 after 24 h) was applied (Figure 5A,C). It was noted that the lowest OD values, i.e., the highest antibacterial activity against *S. mutans* was recorded when 100 µL E(OEO) was applied during the entire experiment (Figure 5B). The results obtained are consistent with those reported in the EOs analysis (Figure 1), in which the most efficient as antibacterial agent against *S. mutans* is OEO.

The results presented in Figure 6 confirm the previous results reported in subchapter 2.3, namely the synergistic effects exerted by the association of eugenol simultaneously with limonene and α-pinene from binary emulsion E (CLEO/BEO). In this case, the OD values decreased from 2.72 after 1 h of incubation to 0.72 after 24 h, which highlights the high antibacterial potential of this emulsion, which increases over time (Figure 6C). In the case of E (OEO/BEO) and E (OEO/CLEO), the association of the limonene majority component in OEO (97.93%) only with α-pinene or eugenol does not lead to a significant increase in the ability to inhibit micellar development over time (Figure 6A,B). With regards to the antibacterial activity determined in the dynamics, an increase in exposure time is observed when binary emulsion E(CLEO/BEO) was used, with the maximum effect (minimum OD value) recorded after 24 h of exposure.

**Figure 7:**
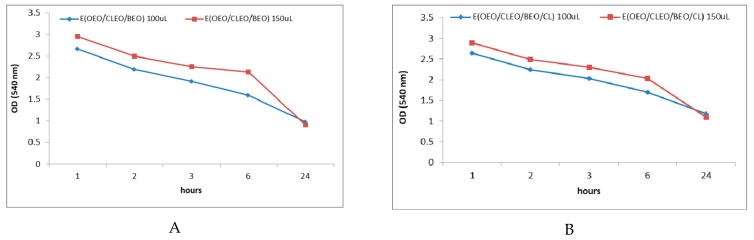
OD values (540 nm) recorded after 1, 2, 3, 6, and 24 h when using tertiary emulsions without chlorhexidine (**A**) and with chlorhexidine (**B**) as antibacterial agents against *S. mutans*.

Figure 7 shows the evolution in time of the development of *S. mutans* treated with the tertiary mixture E (OEO/BEO/CLEO) in the absence and presence of chlorhexidine. The allure of the curve is the same as the inhibition capacity that increases over time both in the absence of chlorhexidine (OD =2.956 after 1 h and decreases to 0.906 after 24 h) (Figure 7A) and in the presence of chlorhexidine (OD=2.899 after 1 h and decreases at 1.093 after 24 h) (Figure 7B). In this case, the previous results regarding the synergistic effects obtained by associating the active principles from EOs are confirmed.

Previous studies highlight the antibacterial effect of natural extracts even at low concentration against *S. mutans* and the possibility to increase the efficacy of the oral hygene practices by incorporating natural extracts into dentifrices [35]. 

*S. mutans* has the ability of rapid lactic acid formation from dietary carbohydrates, mainly sucrose and glucose [36], and as a consequence, the saliva used in our research came from several donors and was not artificial saliva; as such, it contained sucrose and glucose, which improved the bacterial growth, compared to the in vitro tests done in a saliva-free medium.

## 3. Materials and Methods

### 3.1. The GC-MS Characterisation of EOs 

Comercial essential oils (CLEO, CIEO, OEO, and BEO) were purchased from Solaris (SC SOLARIS PLANT SRL, Bucharest, Romania (44°42′38″N 25°99′86″E). Before GC-MS analysis, the oils were diluted 1:10 (*v/v*) with n-hexane (Merck, Darmstadt, Germany, CAS-No:110-54-3).

The chemical characterization of EOs was performed using GS/MS QP 2010Plus (Shimadzu, Kyoto, Japan) equiped with AT WAX 30 m × 0.32 mm × 1 μm capillary column. Helium was used as a carrier gas at a flow rate of 1 mL/min. The program used for the compounds separation was: 40 °C for 1 min, a rate of 5 °C/min to 210 °C for 5 min. Injector and ion source temperatures were 250 °C and 220 °C, respectively. The injection volume was 1 μL at a split ratio of 1:50. The NIST 02 and Wiley 275 libraries spectra library have been used to identify the volatile compounds. The linear retention indices (LRI) were determined in relation to a homologous series of n-alkanes (C8–C24) under the same operating conditions [37].

### 3.2. Microbial Strain and Culture Preparation 

The bacterial strain used in this study was *S. mutans* (ATCC 25175), obtained from the culture collection of the Laboratory of Microbiology in the Interdisciplinary Research Platform within Banat’s “King Michael I of Romania” University of Agricultural Science and Veterinary Medicine Timisoara. In our laboratory, the ATCC strains are maintained at –50 °C. The strain was revived by overnight growth in brain heart infusion (BHI) broth (Oxoid, CM1135), at 37 °C, and, subsequently, passed on BHI Agar, for 24 h at 37 °C. The strain was then diluted with saline solution 4.5‰, at an optical density (OD) of 0.5 McFarland standard (1.5 × 108UFC/ mL); 1 ml of this solution was then suspended in BHI broth 1:30 [38].

### 3.3. Antimicrobial Assay; Optical Density Loss (OD)

McFarland standards can be used to visually approximate the concentration of cells in a suspension. The McFarland scale is CFU/ml specific concentrations and is designed to be used to estimate bacterial concentrations. McFarland standards are generally labeled between 0.5 and 10 and are barium salt suspensions. The spectrophotometric method directly measures the turbidity of the suspension. In visible light, the suspension appears cloudy [39].

A 10^−2^ dilution of the fresh culture of *S. mutans* was used to perform the assay. The resulting suspension was tested using a 96-well, flat-bottomed microdilution plate of a usable volume of 200 μL. Using a Calibra 852 multi-channel pipette, 100 μL of suspension was placed in each well.

Over each suspension well, the oil was directly used, introducing 5, 7, and 10 μL into each well. Plates were covered and left overnight at 37 °C; then, OD was measured at 540 nm using an ELISA reader (BIORAD PR 1100, Hercules, CA, USA). Three-fold tests for all samples were performed. Suspensions of strain in BHI were used as negative control. The MIC of the compound was determined as being the lowest concentration of that compound that inhibited the visible growth of cells.

The same procedure was applied to the emulsions prepared according to the methodology presented below. The used emulsion volume was calculated taking into account the effectiveness of the essential oils analyzed at 50, 100, and 150 μL.

To interpret the results, we calculated:

Micellium grow rate (MGR, %) and micellium inhibition rate (MIR, %) using the formulas:

Micellium grow rate MGR (%) = (OD sample/OD negative control) × 100;

Micellium inhibition rate MIR (%) = 100 − Micellium grow rate MGR (%);

where:

OD sample—optical density at 540 nm for essential oils/emulsion in presence of *S. mutans;*

OD negative control—optical density at 540 nm for *S. mutans* in BHI.

The analytical standards used for testing were: α-pinene (Sigma-Aldrich, CAS Number 7785-70-8, Merck KGaA, Darmstadt, Germany), (R)-(+)-limonene (Sigma-Aldrich, CAS Number 5989-27-5, Merck KGaA, Darmstadt, Germany), and eugenol (Sigma-Aldrich, CAS Number 97-53-0, Merck KGaA, Darmstadt, Germany).

For saliva tests, the experimental model was similar to the in vitro experiment. Sampling of the biological material was performed according to the methodology described by Salli et al. (2017), with the difference that artificial saliva was replaced with saliva from a human donor [40]. Prior to the start of the experiment, the subject gave his written consent for the use of saliva and its inclusion in the experiment. Participation was voluntary and participants were recruited from the interdisciplinary research platform belonging to Banat’s “King Michael I of Romania” University of Agricultural Science and Veterinary Medicine Timisoara staff and were three healthy adult volunteers. Exclusion criteria included chronic or acute illness in the past 3 months, cold or flu symptoms, oral health concerns, and any medication. Participants were asked not to eat, drink, smoke, or use oral hygiene products for 2 h prior to donation. Saliva was collected for 10 min by dribbling into a sterile 50 mL tube. Fresh saliva was collected and immediately used for the experiments. No protease or phosphatase inhibitors were added to the saliva.

### 3.4. Preparation of Emulsion Based on Essential Oils

Emulsions are heterogeneous dispersed systems obtained by mixing two immiscible liquids, a nonpolar component (oil phase) and a polar component (aqueous phase), in the presence of a third component called emulsifier. The emulsifier favors the emulsification and determines the transformation of the two liquids in the discontinuous phase or in the continuous phase, depending on the properties of the two liquids. Depending on the nature of the two phases, there are two types of emulsions: Direct emulsions and reverse emulsions. Direct emulsions are oil-in-water (O/W) type emulsions in which the discontinuous phase is the oil globules dispersed in the aqueous phase.

In our experiment, the preparation of direct emulsions, the emulsifier (lecithin) was dissolved in the aqueous phase (the external phase). In this, it disperses by homogenizing the internal phase (oil). The emulsion was filled with water up to the mass (*m/m*). In 10 ml of distilled water, 6 mg of liquid lecithin (Walmark, 1200 mg) was added and for each emulsion, 500 µL of each selected oil were taken and mixed using a SONICS VCX130 PB 130 Watt Ultrasonic Processor (Newtown, CT, USA) with timer and pulser for 10 min at an amplitude of 98%. 

The natural emulsions with antibacterial activity against *S. mutans* were obtained using essential oils from CLEO, BEO, and OEO; with the addition of emulsifiers, the following emulsifiable formulas were obtained:

- Emulsion E(OEO): Prepared with 500 µL OEO, 6 mg lecithin, 10 mL water;
- Emulsion E(CLEO): Prepared with 500 µL CLEO, 6 mg lecithin, 10 mL water;
- Emulsion E(BEO): Prepared with 500 µL BEO, 6 mg lecithin, 10 mL water;
- Emulsion E(CLEO/BEO): Prepared with 500 µL CLEO+500 µL BEO, 6 mg lecithin, 9.5 mL water;
- Emulsion E(OEO/BEO): Prepared with 500 µL OEO+500 µL BEO, 6 mg lecithin, 9.5 mL water;
- Emulsion E(BEO/CLEO): Prepared with 500 µL BEO+500 µL CLEO, 6 mg lecithin, 9.5 mL water;
- Emulsion E(OEO/CLEO/BEO): Prepared with 500 µL OEO+500 µL CLEO+500 µL BEO, 6 mg lecithin, 9 mL water;
- Emulsion E(OEO/CLEO/BEO) Clorhexidine: Prepared with 500 µL OEO+500 µL CLEO+500 µL BEO, 6 mg lecithin, 1 mL clorhexidine 0.2%, 8 mL water.

### 3.5. Statistical Analysis

The results are presented as the mean ± standard deviation (SD) (*n* = 3). Statistical processing of data was performed using Microsoft Excel 2010 and OriginPro 2018. Significant statistical differences of investigated parameters were determined by *t*-Test: Two-sample assuming equal variances at (*p* < 0.05), after analysis of variance (ANOVA one-way).

## 4. Conclusions

Our in vitro studies on the antibacterial effect of essential oils highlight the synergistic/antagonistic effect of the active principles (limonene, α-pinene, and eugenol) of CLEO, OEO, and BEO in natural preparations, which inhibit/favorsthe development of *S. mutans*. By associating tertiary emulsions based on essential oils CLEO/BEO/OEO or binary (CLEO/BEO) with chlorhexidine, the inhibitory effect is maximized. This mixture is recommended as an antibacterial agent against *S. mutans* developed in the oral cavity. The studies on saliva samples present the antibacterial potential of the emulsifiable formulations, E(CLEO/BEO/OEO), or binary (CLEO/BEO). The antibacterial effect increases over time in the case of emulsions, especially the tertiary one, with the maximum inhibition being recorded 24 h after the treatment. 

The use of essential oils as such, or in emulsifiable form, are viable alternatives as antimicrobial agents, as this is a natural, inexpensive, and effective method for controlling the bacteria involved in oral infections.

Given the proven antimicrobial capacity of essential oils and, in particular, of the oils studied on *S. mutans*, future work must be aimed at understanding the study of the inhibition capacity of essential oils on other bacteria involved in the development of caries and the proliferation of oral infections, as well as testing of other excipients solutions that can potentiate the effect of EOs.

## Figures and Tables

**Table 1:** Chemical composition (% of total) of cloves (CLEO), cinnamon (CIEO), bergamot (BEO), and orange (OEO).

Nr.	Compounds	Type	Retention Time	LRI	% of Total			
					CLEO	CIEO	BEO	OEO
1.	α-pinene	MH	5.58	1013	-	-	32.629	-
2.	camphene	MH	6.62	1057	-	-	0.420	-
3.	β-pinene	MH	7.67	1092	-	-	1.617	-
4.	thujene	MO	7.99	1416	-	-	0.164	-
5.	β-myrcene	MH	9.09	1164	-	-	0.939	1.520
6.	D-limonene	MH	10.15	1189	-	-	49.381	97.930
7.	eucalyptol	MO	10.41	1198	-	-	0.242	-
8.	trans-o-cymene	MH	11.34	1238	-	-	1.162	-
9.	cis-o-cymene	MH	11.43	1241	-	-	3.819	-
10.	4-carene	MH	12.47	1277	-	-	6.345	
11.	butanoic acid hexyl ester β-linalool	- MO	15.550 19.41	1532	- -	- -	0.092 3.020	- 0.550
12.	α -caryophyllene	SH	21.08	1598	5.558	2.052	-	-
13.	β-farnesene	SH	22.68	1664	-	-	0.050	-
14.	α-terpineol	MH	23.44	1695	-	-	0.120	-
15.	cinnamaldehide	MO	27.45	1996	-	0.386	-	-
16.	cinamil-alchool	MO	30.45	2063	-	**88.452**	-	-
17.	cinnamyl-acetate	MO	32.54	2163	-	6.133	-	-
18.	p-eugenol	MO	32.83	2192	**80.106**	2.977	-	-
19.	eugenol acetate	MO	30.627	2277	13.544	-	-	-
20.	chavicol	MO	35.94	2318	0.792	-	-	-
Total of major compounds					100.00	100.0	100.00	100.00
Monoterpene hydrocarbonates (MH)							96.524	99.45
Monoterpene oxygenate (MO)					94.44	97.948	3.426	0.55
Sesquiterpene hydrocarbonates (SH)					5.56	2.052	0.050	-
Sesquiterpene oxygenate (SO)					-	-	-	-
